# Regulation of p53 by Jagged1 Contributes to Angiotensin II-Induced Impairment of Myocardial Angiogenesis

**DOI:** 10.1371/journal.pone.0076529

**Published:** 2013-10-03

**Authors:** Aili Guan, Hui Gong, Yong Ye, Jianguo Jia, Guoping Zhang, Bingyu Li, Chunjie Yang, Sanli Qian, Aijun Sun, Ruizhen Chen, Junbo Ge, Yunzeng Zou

**Affiliations:** 1 Shanghai Institute of Cardiovascular Diseases, Zhongshan Hospital and Institutes of Biomedical Sciences, Fudan University, Shanghai, China; 2 Department of Cardiology, Qingdao Municipal Hospital, Qingdao, China; Georgia Regents University, United States of America

## Abstract

Angiotensin II (AngII) is a major contributor to the development of heart failure, however, the molecular and cellular mechanisms still remain elucidative. Inadequate angiogenesis in myocardium leads to transition from cardiac hypertrophy to dysfunction, this study was therefore conducted to examine the effects of AngII on myocardial angiogenesis and the underlying mechanisms. AngII treatment significantly impaired angiogenetic responses, which were determined by counting the capillaries either in matrigel formed by cultured cardiac microvascular endothelial cells (CMVECs) or in myocardium of mice and by measuring the *in vitro* and *in vivo* production of VEGF proteins, and stimulated accumulation and phosphorylation of cytosolic p53 which led to increases in phosphorylated p53 and decreases of hypoxia inducible factor (Hif-1) in nucleus. All of these cellular and molecular events induced by AngII in CEMCs and hearts of mice were largely reduced by a p53 inhibitor, pifithrin-α (PFT-α). Interestingly, AngII stimulated the upregulation of Jagged1, a ligand of Notch, but it didn’t affect the expression of Delta-like 4 (Dll-4), another ligand of Notch. Inhibition of p53 by PFT-α partly abolished this effect of AngII. Further experiments showed that knockdown ofJagged1 by addition of siRNA to cultured CMVECs dramatically declined AngII-stimulated accumulation and phosphorylation of p53 in cytosol, upregulation of phosphorylated p53 and downregulation of Hif-1 expression in nucleus, decrease of VEGF production and impairment of capillary-like tube formation by the cells. Our data collectively suggest that AngII impairs myocardial angiogenetic responses through p53-dependent downregulation of Hif-1 which is regulated by Jagged1/Notch1 signaling*.*

## Introduction

Adaptive formation of angiogenesis has been reported to be significantly involved in the mechanisms by which cardiac hypertrophy maintains compensatory state during pressure overload [1]. The increase in the production and secretion of angiogenetic growth factors, such as VEGF, in the hypertrophied heart is responsible for the enhanced angiogenesis [1,2]. Inadequate angiogenesis due to the disorders of angiogenetic factors during the development of cardiac hypertrophy is the main reason for that the adaptive cardiac hypertrophy transmits to heart failure [1]. Activation of renin-angiotensin system (RAS) participates greatly in the development of cardiac hypertrophy after pressure overload [3]. Angiotensin II (AngII), the main component of RAS, exerts effects not only on formation of cardiomyocyte hypertrophy and cardiac fibrosis but also on dysregulation of myocardial angiogenesis [4]. AngII infusion may reduce the density of cardiac vessels and impair the angiogenetic capacity of aorta and coronary artery rings in rats [5]. However, the potential mechanism of how AngII impairs myocardial angiogenesis remains largely elucidative, although the molecular mechanisms by which AngII induces cardiomyocyte hypertrophy and growth of cardiac fibroblasts have been extensively investigated,

The report by Sano M et al indicated that sustained pressure overload induces an accumulation of p53 that inhibits hypoxia-inducing transcription factor (Hif-1) activity, thereby impairing cardiac angiogenesis during the development of cardiac hypertrophy [1,6]. Our recent study showed that AngII at a certain concentration exerted an inhibitory effect on the formation of vasculatures by cultured cardiac microvascular endothelial cells (CMVECs). However, whether AngII could directly inhibit cardiac angiogenesis through p53-regulated inhibition of Hif-1 waits to be clarified. On the other hand, the Notch pathway is a highly conserved signaling system that regulates a diversity of biological processes of cells. Recent studies have shown that Notch signaling is involved in the regulation of angiogenesis [7]. It has been shown that the Notch ligand Jagged1 is a potent proangiogenetic regulator, whereas binding of the Notch receptor to its another ligand Delta-like 4 (Dll-4) inhibits angiogenesis by endothelial tip cells [7]. However, the roles of Notch pathway in AngII-induced impairment of cardiac angiogenesis are not clear.

In this study, we used AngII to induce the impairment of angiogenesis in cultured CMVECs and heart tissue, and investigated the roles of p53 and Notch ligands Jagged1 and Dll-4 in regulation of the angiogenetic sickness. Similar to what we have shown in pressure overload-induced impairment of angiogenesis [1], accumulation and activation of p53 induced inhibition of Hif-1, by which AngII impaired ability of vasculature formation by CMVECs. Jagged1, but not Dll-4, was related to the AngII-stimulated accumulation and phosphorylation of p53. Together, they resulted in the impairment of cardiac angiogenesis.

## Methods

### Cell culture

Primary CMVECs were obtained from the heart of male Wistar rats (8-9 weeks old) by the method of planting myocardial tissues [8,9], and were cultured in high-glucose Dulbecco’s modified Eagle’s medium (DMEM) (Gibco) containing 20% fetal bovine serum (FBS, Hyclone), 100 U/ml penicillin and 100 U/ml streptomycin (Sigma-Aldrich). The 2-3 passages of rat CMVECs were seeded on a 6-well plate at 5×10^5^/well and cultured for 24 hours. Before the treatments, CMVECs were cultured for 24 hours in serum-free DMEM. To induce angiogenetic injury, CMVECs were treated with 1µM AngII for 18 hours [10]. At the end of treatments, CMVECs were collected for further analyses of capillary-like tube formation, Western blotting, real-time RT-PCR and enzyme-linked immunosorbent assay (ELISA). The human umbilical vein endothelial cell (HUVECs) were purchased from CELL BANK of Chinese Academy of Sciences, and were maintained in DMEM (Gibco) supplemented with 10% FBS and 100 U/ml penicillin-streptomycin mixture (Sigma-Aldrich) at 37 °C and 5% CO_2_ in a humidified chamber. The HUVECs were transfected with luciferase plasmid and collected for Dual Luciferase Assay.

### Animals

Eight-week-old male C57BL/6 mice were purchased from Shanghai Animal Administration Center (Shanghai, China). AngII (200 ng/kg/min, Sigma-Aldrich) was continuously administered to mice for 2 weeks by Alzet micro-osmotic pumps (DURECT Corporation) implanted subcutaneously [10]. PFT-α (Sigma-Aldrich) was dissolved in dimethyl sulfoxide (DMSO), and the dilution was injected into mice intraperitoneally with a 3.0 mg/kg dose one day [11] before AngII infusion and then was injected in a similar dose once every 3 days during AngII infusion. At the end of experiments, mice were subjected to the echocardiography, noninvasive blood pressure (NIBP) measurements and analyses for myocardial immunohistochemistry and Western blot. All protocols were approved by the Animal Care and Use Committee of Fudan University, and in compliance with “Guidelines for the Care and Use of Laboratory Animals” published by the National Academy Press (NIH Publication No. 85–23, revised 1996).

### Capillary-like tube formation

200 µl growth factor-reduced Matrigel matrix (BD) was homogenized, layered into a 24-well plate on a cooled planar surface and allowed to solidify at 37 °C [12]. CMVECs were detached with trypsin-EDTA, resuspended in high-glucose DMEM containing 1% FBS and added by AngII (1 µM) [10] with or without PFT-α (50µM) [13,14], or vehicle. The suspension was seeded into plates already embedded by matrigel at 5×10^4^ cells/well and cultivated at 37 °C atmosphere with 5% CO2 /95% air for 18 hours. Capillary-like tube formation was analyzed as our previous study [6]. Images of cultured CMVECs were taken at 50× magnification with a digital output camera attached to an inverted phase-contrast microscope (Leica, Germany), five random view-fields per well were counted to analyze the relative number of capillary-like tubes visible (Figure S1), and the values averaged. During every experiment, three wells per group were examined. The data were obtained from 15 independent experiments. The representative photograph of capillary-like tube formation were shown at 100× magnification.

### Western blot analysis

Total proteins, nuclear proteins or cytoplasmic proteins were isolated from left ventricular tissues or culture cells by the methods [6]. Isolated proteins (50-100 µg) were size-fractionated by SDS–PAGE and transferred to polyvinylidene difluoride membrane. And then the membranes were incubated with primary antibodies followed by incubation with a HRP-conjugated secondary antibody (Jackson). All antibodies used here were: phosphor-p53 (pS392), p53 and Hif-1α (EPITOMICS); rabbit polyclonal to TFIIB or to Jagged1 (Abcam); β-actin (Kangchen, China).

### Immunohistochemistry

Left ventricles (LV) were fixed in 10% formalin, embedded in paraffinor frozen in cryomolds, sectioned at 4-µm thickness and stained with an anti-CD31 antibody (Santa Cruz). For measurement, five random high-power fields from each section were chosen and quantified in a blinded manner. CD31 positive vasculatures were measured in 5 sections from each LV and the mean value was expressed.

### siRNA and in vitro transfection

siRNAs targeting ratJagged1 and human-Jagged1were obtained from Shanghai GenePharma and RiboBio Company (China), respectively, and three sequences of targeting-rat Jagged1 or human Jagged1 were: 1^#^, 2^#^, 3^#^ respectively. Fugene transfection reagent (Roche) and siRNAs were mixed and kept still for 20 min at room temperature, and then the mixtures were added to CMVECs or HUVECs cultured in 6-well plates for transfection. The scramble sequences were also transfected and used as controls.

### ELISA assay

Supernates of cultured CMVECs were collected and examined by Quantikine® (R&D Systems) for VEGF immunoassay following the manufacturer’s instructions. In brief, supernates were added to VEGF-antibody coated plate and incubated at room temperature for 2 hours. After adding stop solution, we read absorbance of each microwell on a spectrophotometer using 450nm as the primary wavelength.

### Cell culture luciferase assays

After siRNA-Jagged1 or siRNA-scramble transfection for 24 hours, human umbilical vein endothelial cells (HUVEC) were transfected with either pcDNA3- Hif-1-Luc (Addgene, from Dr. William Kaelin) [15] or pRBPj-luc (Addgene, from Dr. Nicholas Gaiano) [16] and β-galactosidase expression plasmid (Applied Biosystems) by using Fugene transfection reagent according to the manufacturer’s instructions. The next day, cells were treated with Ang II (1µm) followed by PFT-α (50µM) or DMSO incubation for 30 min. Forty-eight hours later, luciferase activity of cells extracts was measured with the Dual Luciferase Assay System (Applied Biosystems) according to the manufacturer’s instructions and a Berthold Technologies luminometer.

### Statistical analysis

All the data were presented as mean ± S.E.M. and were analyzed using One-Way ANOVA tests followed by Least-Significance-Difference (LSD) method for multiple means comparison or using Independent-Samples *t*-tests for two means comparison. A two-tailed *p*-value of < 0.05 was considered statistically significant. All statistical analysis was performed by SPSS 16.0 for windows.

## Results

### 1: AngII induced impairment of angiogenetic responses and enhancement of p53 accumulation and phosphorylation in cultured CMVECs

**Figure 1:**
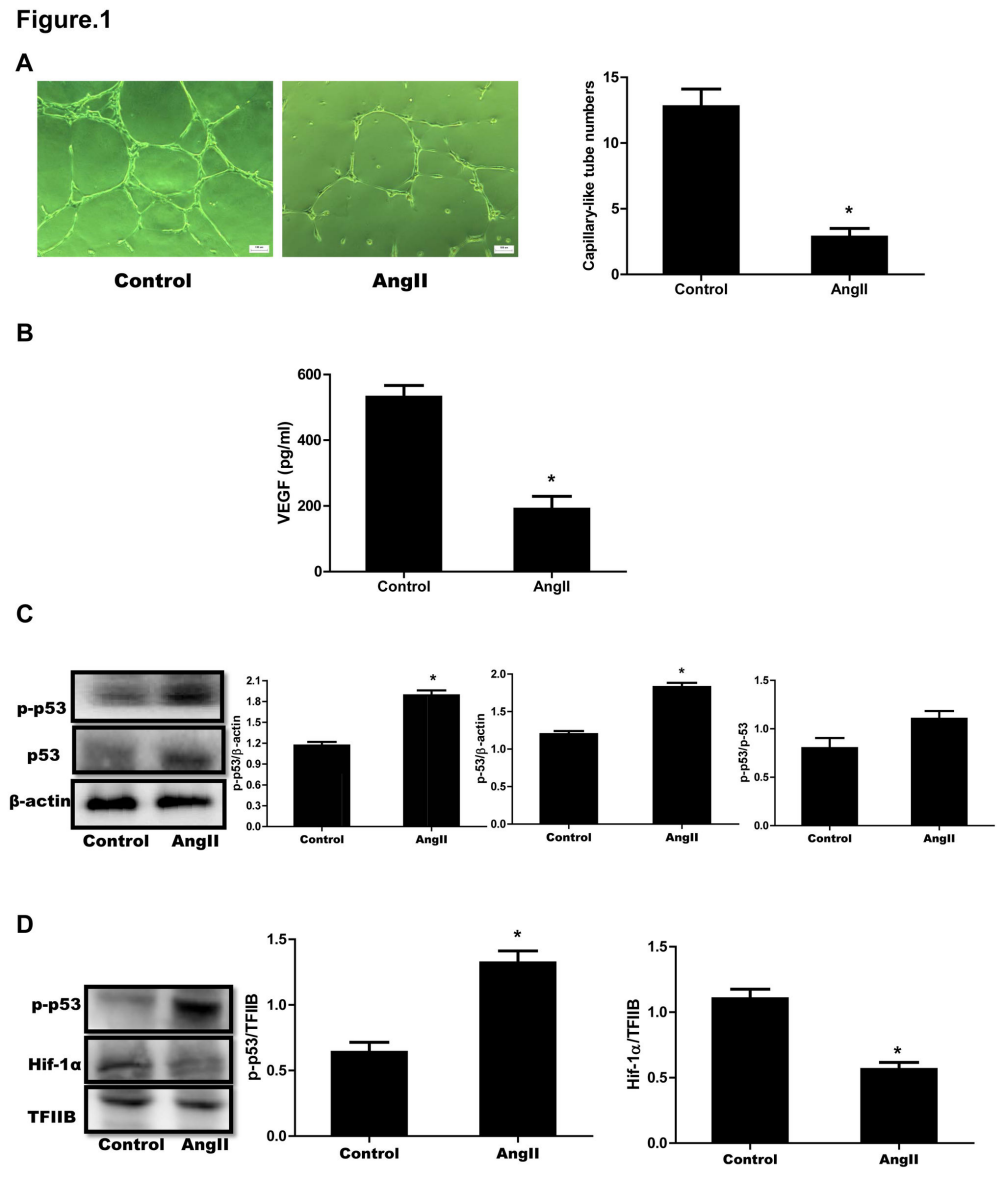
AngII-induced impairment of angiogenetic responses and accumulation and phosphorylation of p53 in cultured CMVECs. (**A**) Formation of capillary-like tubes. CMVECs were seeded onto the matrigel in plates containing 1 µM AngII (AngII) or vehicle (Control). Eighteen hours later, the formation of capillary-like tubes was observed under an optical microscope. *Left* *panel*: Representative photomicrographs of capillary-like tubes (scale bar: 100 µm). *Right* *panel*: Quantitative analysis for capillary-like tube formation. Capillary-like tubes were counted in randomly selected 5 fields for each plate. Data are expressed as mean ± S.E.M. obtained from 15 independent experiments (n=15). (**B**) Measurement of VEGF protein levels in culture medium. Cultured CMVECs were incubated with AngII (1 µM) or vehicle (Control) for 18 hours. Culture medium was collected and VEGF proteins were measured by ELISA. Data are expressed as mean ± S.E.M. obtained from 5 independent experiments (n=5). (**C**) Cytosolic p53 and p-p53 protein expression. CMVECs were treated as described under (B) and then collected and lysed for Western blot analyses of p53 and p-p53. β-actin was used as a loading control. (**D**) Nucleus p-p53 and Hif-1α expression. Nucleus proteins isolated from CMVECs treated as described under (B) were subjected to Western blot analyses for p-p53 and Hif-1α. TFIIB was used as a loading control. Representative immunoblots are shown. The expression of p53, p-p53 or Hif-1α was quantified as folds of β-actin, p53 or TFIIB, respectively. Data represent mean ± S.E.M. obtained from 3 independent experiments (n=3). ^*^ *p* < 0.05 *vs* Control.

We first confirmed the effects of AngII on capillary-like tube formation by cultured CMVECs. We selected 1µM Ang II for the following experiment according our previous study [12]. Treatment with AngII (1µM) for 18 hours resulted in a significant decrease of the number of capillary like-tubes formed by CMVECs (Figure 1A). We also examined the secretion of VEGF, the important angiogenesis-promoting factor, by cultured CMVECs. The VEGF level was significantly lower in culture medium of AngII-treated CMVECs than in that of vehicle treated ones (Figure 1B). In an earlier report, Sano M et al. showed that accumulation of p53 inhibited Hif-1 activity and impaired cardiac angiogenesis during pressure overload [6]. The present study showed that AngII also enhanced p53 accumulation and phosphorylation at serine-392 in cytosol of CMVECs (Figure 1C). Additionally, phosphorylated p53 (p-p53) has been indicated to accumulate in nucleus of the cells and bind to target genes to function in response to stresses [17]. We therefore detected the levels of p-p53 and Hif-1α in nucleus of CMVECs after stimulated by AngII. The results obviously revealed that the p-p53 was elevated and the Hif-1α was downregulated in nucleus of AngII-treated CMVECs as compared to vehicle-treated cells (Figure 1D).

### 2: Inhibition of p53 improved AngII-induced impairment of angiogenetic responses in cultured CMVECs

**Figure 2:**
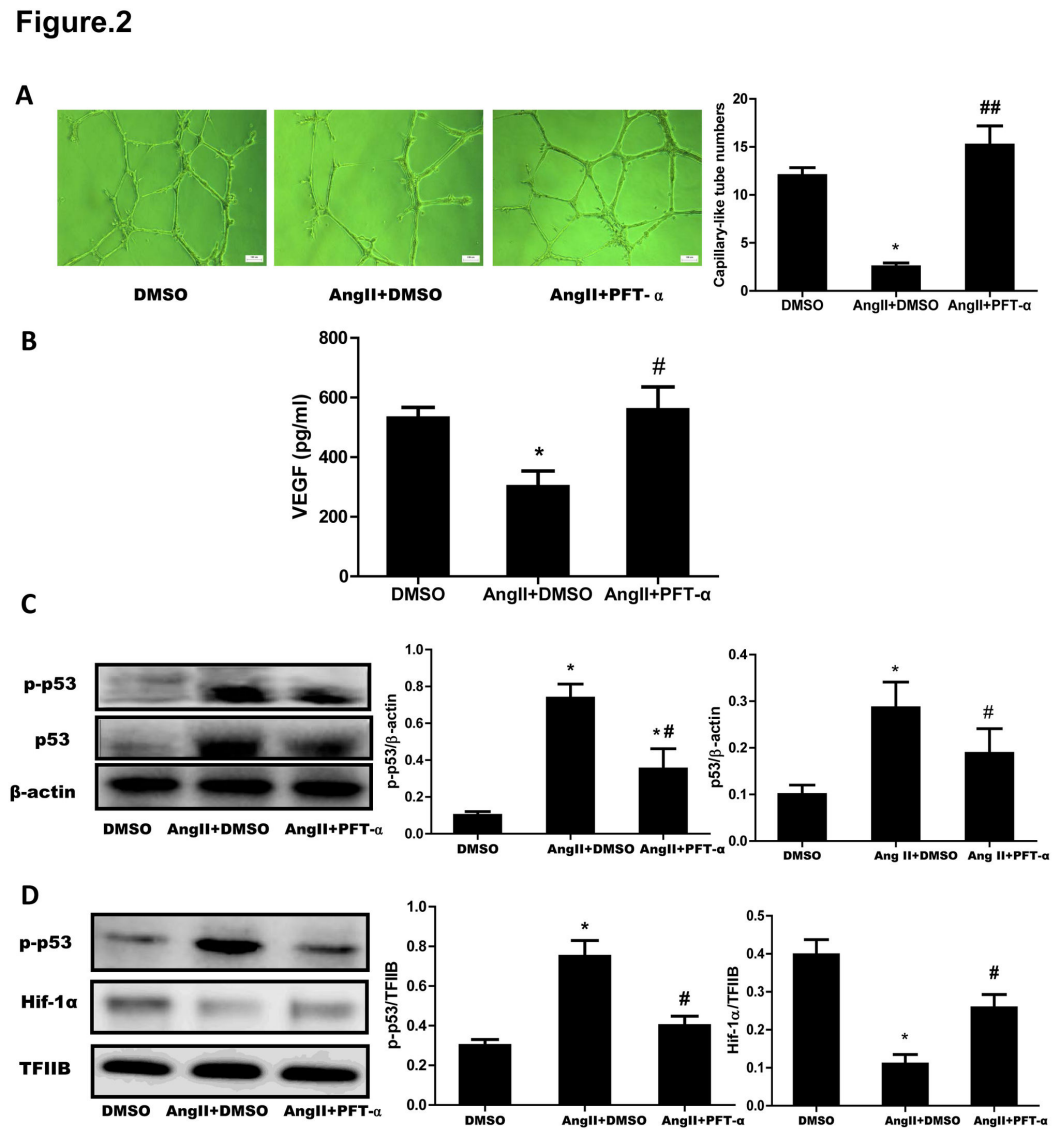
Effect of p53 inhibitor on AngII-induced impairment of angiogenetic responses in cultured CMVECs. (**A**) Formation of capillary-like tubes. CMVECs treated with PFT-α or with vehicle (DMSO) for 30 min were seeded onto the matrigel in plates containing 1 µM AngII with or withour PFT-α. CMVECs treated with DMSO and seeded onto the matrigel without AngII served as the control (DMSO). Eighteen hours later, the formation of capillary-like tubes was observed under an optical microscope. *Left* *panel*: Representative photomicrographs of capillary-like tubes (scale bar: 100 µm). *Right* *panel*: Quantitative analysis for capillary-like tube formation. Capillary-like tubes were counted in randomly selected 5 fields for each plate. Data are expressed as mean ± S.E.M. obtained from 15 independent experiments (n=15). (**B**) Measurement of VEGF protein levels in culture medium. CMVECs were pretreated with PFT-α or DMSO for 30 min and then incubated with AngII (1 µM) for 18 hours. CMVECs treated with DMSO but without AngII served as the control (DMSO). Culture medium was collected and VEGF proteins were measured by ELISA. Data are expressed as mean ± S.E.M. obtained from 5 independent experiments (n=5). (**C**) Cytosolic p53 and p-p53 protein expression. CMVECs treated as described under (B) were collected and lysed for Western blot analyses for p53 and p-p53. β-Actin expression served as a loading control. (**D**) Nucleus p-p53 and Hif-1α expression. Nucleus proteins isolated from CMVECs treated as described under (B) were subjected to Western blot analyses for p-p53 and Hif-1α. TFIIB was used as a loading control. Representative immunoblots are shown. The expression of p53, p-p53 or Hif-1α was quantified as folds of β-actin or of TFIIB, respectively. Data represent mean ± S.E.M. obtained from 3 independent experiments (n=3). ^*^ *p* < 0.05 *vs* DMSO; ^#^ *p* < 0.05 *vs* AngII+DMSO.

To ask the role of p53 in the effect of AngII on angiogenesis, we treated CMVECs with PFT-α (50 µM), a selective inhibitor for p53 transcriptional activity as previous study [13,14]. AngII-induced decrease of the capillary-like tube formation by CMVECs was significantly improved by treatment with PFT-α (Figure 2A). And after stimulation with AngII, the VEGF level in culture medium collected from PFT-α-treated CMVECs was higher than in that collected from vehicle (DMSO)-treated ones (Figure 2B). Western blot analysis confirmed that both the accumulation and phosphorylation of p53 either in cytosol or in nucleus of CMVECs induced by AngII were simultaneously inhibited by PFT-α treatment (Figure 2C, 2D). Also, PFT-αtreatment partially reversed AngII-impaired expression of Hif-1α in nucleus of CMVECs (Figure 2D). We further detected Hif-1 activity by transfecting Hif-1-luc report gene in HUVECs for the poor luciferase induction in CMVECs, PFT-α abolished Ang II-induced the upregulation of Hif-1 activity (Figure S2). These results indicated that AngII-induced impairment of angiogenesis in cultured CMVECs is dependent on p53 function.

### 3: p53 functioned on AngII-induced impairment of cardiac angiogenesis in mice

**Figure 3:**
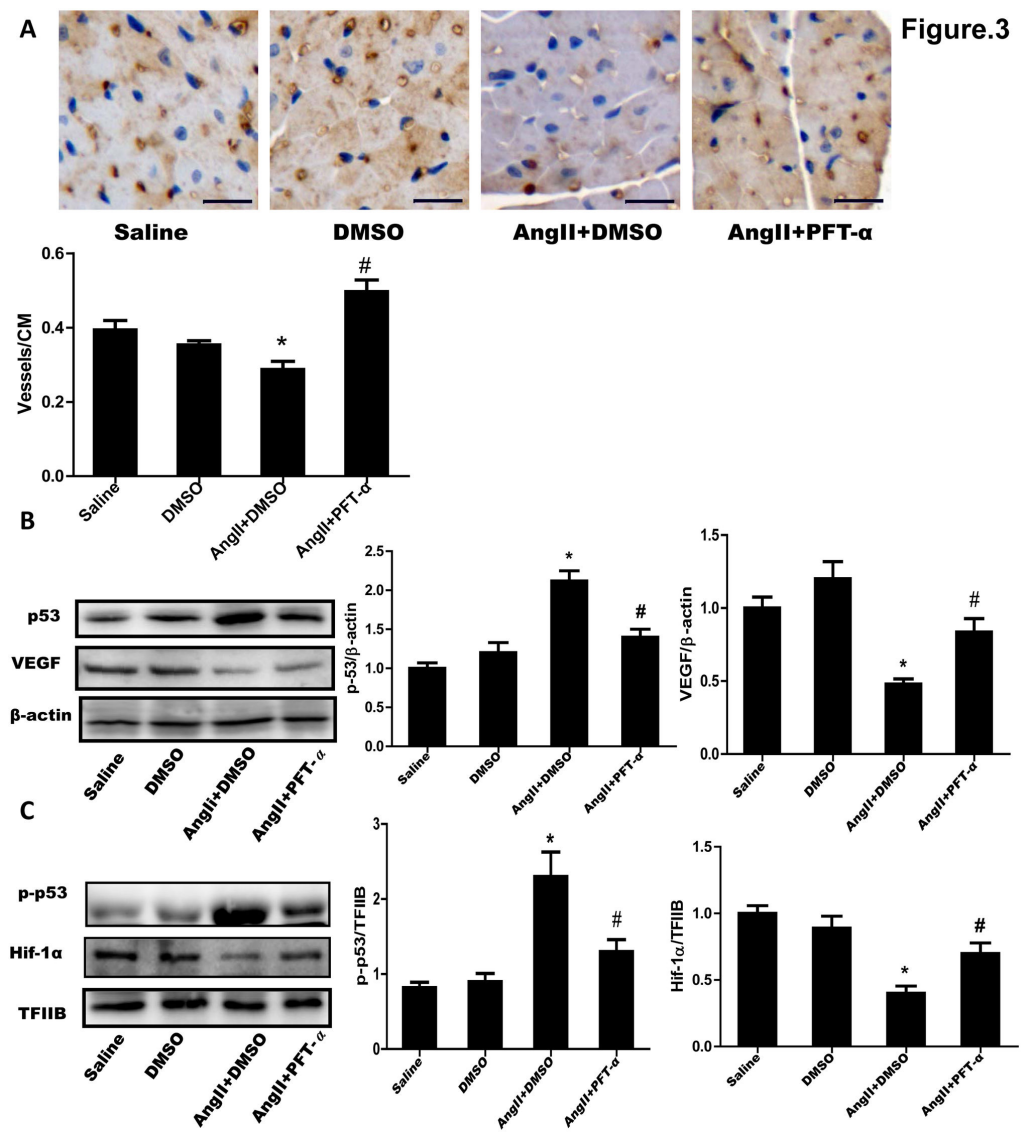
Effect of p53 inhibitor on AngII-induced impairment of myocardial angiogenesis in mice. AngII (200 ng/kg/min) or saline was subcutaneously infused to mice for 2 weeks by Alzet micro-osmotic pumps. PFT-α (3.0 mg/kg) or DMSO was injected into mice intraperitoneally one day before AngII or saline infusion and then was injected in a similar dose once every 3 days during the infusion. (**A**) Immunostaining for myocardial CD31. Representative photographs from left ventricle (LV) are shown (scale bar: 20µm). Brown color indicates CD31-positive capillaries. CD31-positive vasculatures were expressed as the numbers per cm^2^ area in LV wall. Data are shown as mean ± S.E.M. from 6 hearts. (**B**) Cytosolic p53 and VEGF expression in LV tissue. Total proteins from LV tissues were subjected to Western blotting. β-Actin was used as a loading control. (**C**) Nucleus p-p53 and Hif-1α expression. Nucleus proteins extracted from LV tissues were subjected to Western blotting. TFIIB was used as a loading control. Representative immunoblots are shown. The expression of p53, VEGF, p-p53 or Hif-1α was quantified as folds of β-actin or of TFIIB, respectively. Data are expressed as mean ±S.E.M. obtained from 3 independent experiments. ^*^ *p* < 0.05 *vs* Saline; ^#^ *p* < 0.05 *vs* AngII+DMSO.

We further confirmed the role of p53 in AngII-induced dysfunction of myocardial angiogenesis in mice. AngII (200 ng/kg/min) or saline was continuously administered to mice through an osmotic minipump for 2 weeks as our previous study [10]. The blood pressure was not affected by the AngII administration at this dosage (Figure S3A, S3B). Echocardiographic and histological analyses showed that the AngII infusion induced a significant cardiac hypertrophy (Figure S3C-3H). Immunohistochemistry analysis for CD31 positive cells in myocardium revealed that AngII administration significantly reduced the densities of CD31-positive vasculatures and that the reduction of vasculatures was abrogated by treatment with PFT-α (3.0 mg/kg) [18] but not by vehicle treatment (DMSO) (Figure 3A). At the same time, PFT-α treatment obviously ameliorated AngII-induced cardiac hypertrophy and dysfunction (Figure S3C-3H). Furthermore, AngII infusion significantly stimulated accumulation of cytosolic p53 and nucleus p-p53, and downregulated VEGF protein production and nucleus Hif-1α expression detected by Western blotting. However, all these events observed during AngII infusion were declined by PFT-α but not by DMSO treatment in myocardium of the mice (Figure 3B, 3C). These *in vivo* results were consistent with those obtained from the *in vitro* experiments*.*

### 4: Expression of Jagged1, but not that of Dll-4, was regulated by p53 both in AngII-stimulated CMVECs and AngII-infused myocardium of mice

**Figure 4:**
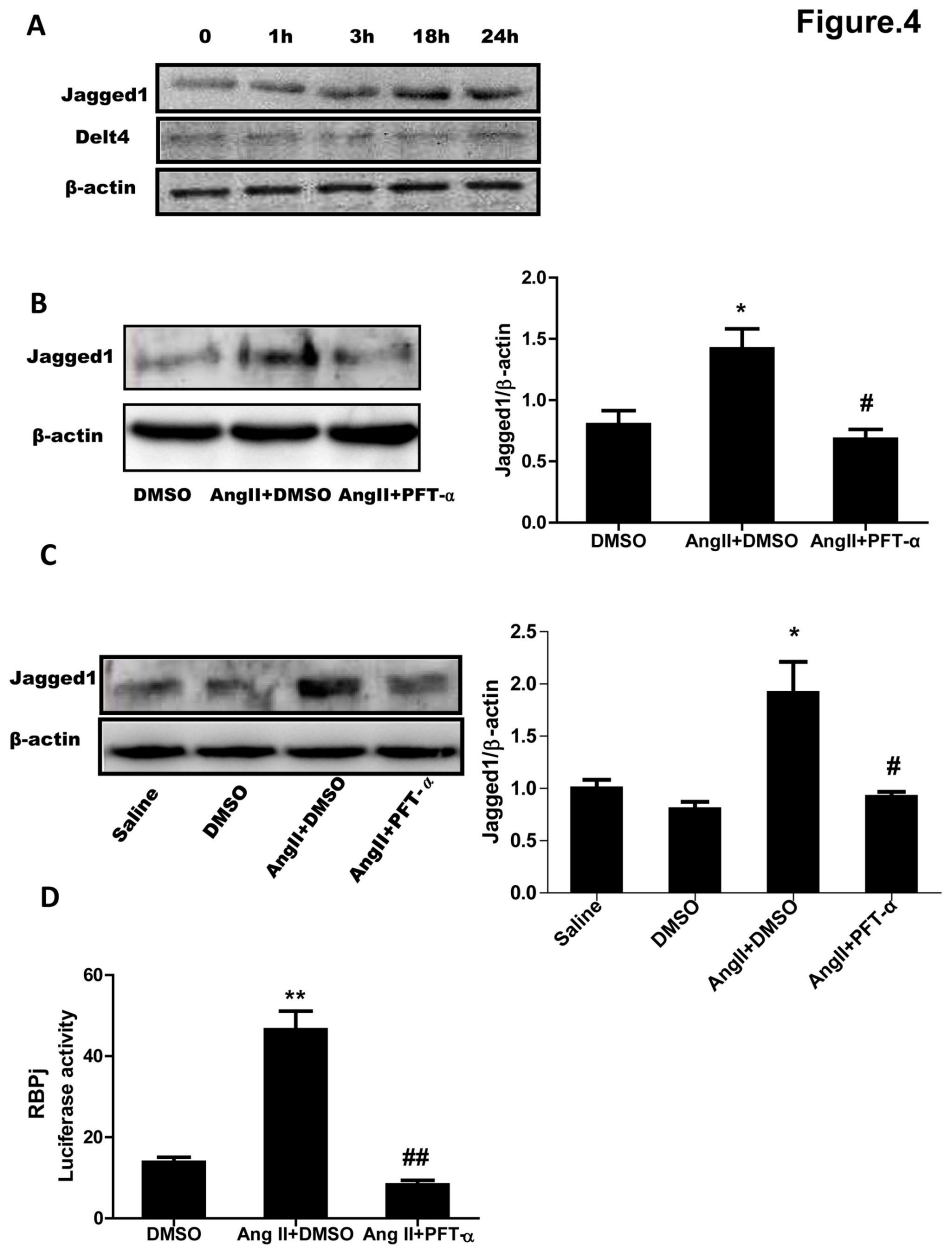
p53-dependent upregulation of Jagged1 expression induced by AngII. (**A**) Expression of Jagged1 and Dll-4 after AngII stimulation. Cultured CMVECs were stimulated with AngII (1 µM) for the indicated times. Jagged1 and Dll-4 expressions were detected by Western blotting. β-Actin was used as a loading control. Representative immunoblots from 3 independent experiments are shown. (**B**) Effects of p53 inhibitor on Jagged1 expression in CMVECs. CMVECs were pretreated with PFT-α or vehicle (DMSO) for 30 min and then stimulated with AngII (1 µM) or Ang II plus PFT-α for 18 hours. CMVECs treated with DMSO but without AngII served as the control (DMSO). Jagged1 expression was detected by Western blotting. β-Actin was used as a loading control. Representative immunoblots are shown. The expression of Jagged1 was quantified as folds of β-actin. Data are expressed as mean ±S.E.M. obtained from 3 independent experiments. (**C**) Effects of p53 inhibitor on Jagged1 expression in hearts of mice. Mice were treated by the method described under Figure 3. LV tissues were subjected to Western blot analysis for Jagged1 expression. β-Actin expression served as a loading control. Representative immunoblots are shown. The expression of Jagged1 was quantified as folds of β-actin. Data are expressed as mean ±S.E.M. obtained from 6 mice. (**D**) Effects of p53 inhibitor on RBPj –luc activity. HUVECs were transfected with rRBPj-Luc construct together with β-gal expression plasmid for 48h, after 30 min of PFT-α pretreatment, the cells were treated with Ang II for 24 hour and subjected for Luc assays. PFT-α: p53 inhibitor. Data are expressed as mean ±S.E.M. obtained from 3 independent experiments. ^*^ *p* < 0.05 ^**^ *p* < 0.01 *vs* DMSO or Saline; ^#^ *p* < 0.05 ^# #^ *p* < 0.01 *vs* AngII+DMSO.

Since Notch ligands Jagged1 and Dll-4 have been shown to be involved in angiogenetic responses [7], we therefore defined the role of them in the mechanism by which AngII induces impairment of angiogenesis. Jagged1 protein expression was increased in a time-dependent manner and peaked at 18 hours after AngII treatment, but AngII didn’t affect the expression of Dll-4 in cultured CMVECs (Figure 4A), suggesting that Jagged1 but not Dll-4 was involved in AngII-stimulated signaling in CMVECs. In order to ask how Jagged1 was involved, we treated CMVECs and mice with PFT-α and detected Jagged1 expression after stimulation or infusion with AngII. Western blot analysis showed that the upregulation of Jagged1 either in cultured CMVECs (Figure 4B) or in myocardium of mice (Figure 4C) induced by AngII were both suppressed by PFT-α but not by DMSO treatment. It has been reported that *Jag1-Notch-Rbpj* signaling is critical for mouse lens progenitor cell growth [19]. To examine the p53 role in *Rbpj* signaling, we transfected HUVECs with RBPj (CBF1)-luc reporter system in which Luc transcription is controlled by the canonical CBF1/RBPj responsive element. The result showed that PFT-α almost abolished Ang II-induced the upregulation of RBPj-luc activity (Figure 4D).

### 5: Inhibition of Jagged1 weakened accumulation and phosphorylation of p53 and improved angiogenetic disorders of CMVECs induced by AngII

**Figure 5:**
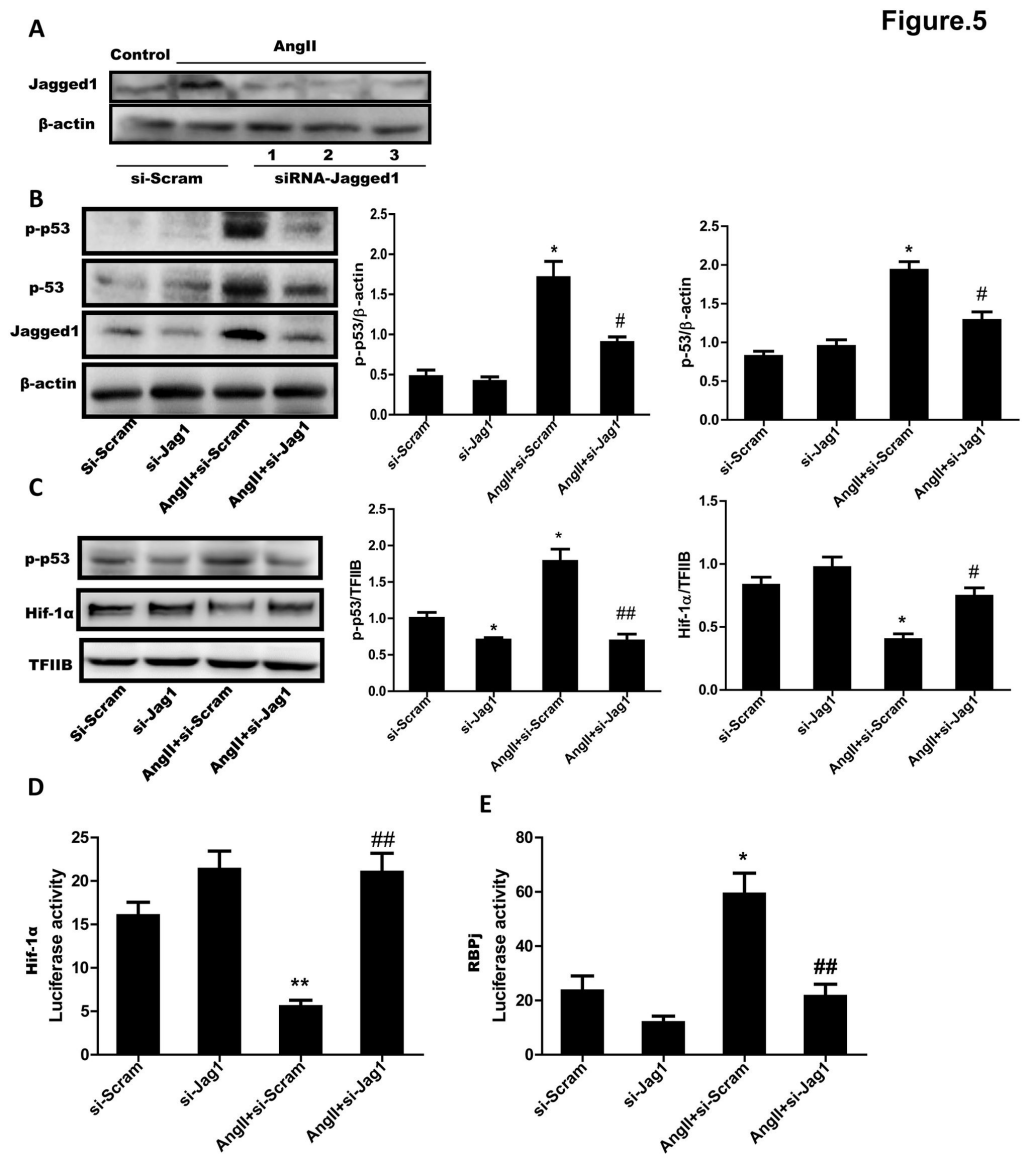
Effect of Jagged1 siRNA on AngII-induced accumulation and phosphorylation of p53 and dowregulation of Hif-1α in CMVECs. (**A**) Downregulation of Jagged1 by siRNA in CMVECs. Three sequences of siRNA of Jagged1 (siRNA-Jagged1, 1 through 3) or scramble RNA were transfected to cultured CMVECs and then the cells were incubated with (AngII) or without (Cont) AngII (1 µM) for 18 hours. Jagged1 expression was detected by Western blotting. β-Actin expression served as a loading control. Representative immunoblots from 3 independent experiments are shown. (**B**) Inhibition of cytosolic p53 and p-p53 expression by siRNA of Jagged1. CMVECs transfected by #2 sequence of Jagged1 siRNA (si-Jag1) or scramble RNA (Scram) were stimulated with or without AngII (1µM) for 18 hours. Total cell lysate was subjected to Western blotting. β-Actin was used as a loading control. (**C**) Effect of Jagged1 siRNA on nucleus p-p53 and Hif-1α expression. Nucleus proteins isolated from CMVECs treated as described under (B) were subjected to Western blot analyses. TFIIB expression served as a loading control. Representative immunoblots are shown. The expression of p53, p-p53 or Hif-1α was quantified as folds of β-actin or of TFIIB, respectively. (**D**) Effect of Jagged1 siRNA on Hif-1 activity. (**E**) Effect of Jagged1 siRNA on notch/RBPj activity. HUVECs were transfected with Hif-1-luc or RBPj-Luc construct together with β-gal expression plasmid following by si-Jag1 transfection. 24 hour later, the cells were treated with Ang II for 24 hour and subjected for Luc assays. Data represent mean ± S.E.M. obtained from 3 independent experiments (n=3). ^*^ *p* < 0.05, ^**^ *p* < 0.01 *vs* Scram; ^#^ *p* < 0.05, ^# #^ *p* < 0.01 *vs* AngII+Scram.

Although the expression of Jagged1 stimulated by AngII was regulated by p53 in cultured CMVECs, the interrelation of them, that is, whether Jagged1 might regulate p53 was not answered. We thus finally examined the role of Jagged1 in AngII-induced accumulation and phosphorylation of p53 and impairment of angiogenesis in cultured CMVECs by using of the siRNA of Jagged1. As shown in Figure 5A, addition of any one of the three siRNA sequences (1 through 3) of Jagged1 induced a sufficient knockdown (>50%) of Jagged1 protein expression in CMVECs elevated by AngII incubation. We chose the number 2 of siRNA for the following experiments. Western blot analysis showed that not only the accumulation and the phosphorylation of p53 in cytosol of CMVECs but also the phosphorylated p53 and the downregulated Hif-1α in nucleus of the cells induced by AngII stimulation were all significantly reversed by the siRNA of Jagged1 (Figure 5B, 5C). We then detected Hif-1 activity by transfecting Hif-1-luc report gene in HUVECs for the poor luciferase induction in CMVECs. Western blot analysis showed that siRNA-2 could effectively knock down Jagged1 expression in HUVECs (Figure S4). So we used siRNA-2 for the following experiment. The result indicated that Ang II greatly inhibited the activity of Hif-1, but si-Jagged1 almost abolished the effects (Figure 5D). To examine whether Ang II stimulates endogenous notch/RBPj mediated activity by the upregulation of Jagged 1 during anti-angiogenetic responses, we transfected HUVECs with RBPj (CBF1)-luc reporter system. The RBPj activity in Ang II-treated-HUVECs was 3-fold higher than in untreated HUVECs, and it was greatly inhibited by si-Jagged1 (Figure 5E).

**Figure 6:**
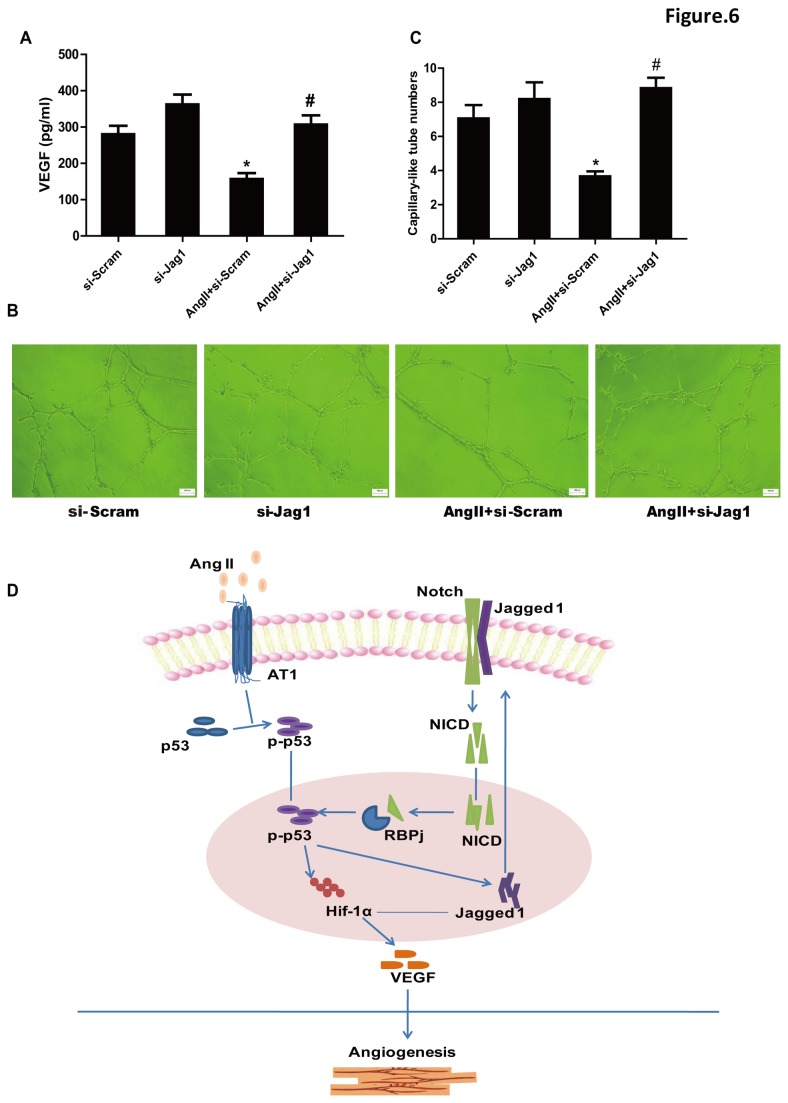
Effect of Jagged1 siRNA on AngII-induced impairment of VEGF production and capillary-like tube formation of CMVECs. (**A**) VEGF production. Cultured CMVECs were treated as described under Figure 5B. Culture medium was collected and VEGF proteins were measured by ELISA. Data are expressed as mean ± S.E.M. obtained from 3 independent experiments (n=3). (**B**) Capillary-like tube formation. CMVECs transfected with #2 siRNA sequence of Jagged1 (si-Jag1) or scramble RNA (Scram) were seeded onto the matrigel in plates containing 1µM AngII or not. Eighteen hours later, the formation of capillary-like tubes was observed under an optical microscope. Representative photographs are shown (scale bar: 100µm). (**C**) Quantitative analysis for capillary-like tube formation. Capillary-like tubes were counted in randomly selected 5 fields for each plate. Data are expressed as mean ± S.E.M. obtained from 5 independent experiments (n=5). ^*^ *p* < 0.05 *vs* Scram; ^#^ *p* < 0.05 *vs* AngII+Scram. (**D**) Schematic diagram of Ang II-induced impairment of myocardial angiogenesis through regulation of p53 by Jagged1.

To confirm whether the regulation of p53 by Jagged1 functions in AngII-impaired angiogenesis, the secretion of VEGF and the formation of capillary like-tubes by cultured CMVECs were analyzed. The amounts of VEGF in culture medium assayed using ELISA methods and the numbers of capillary like-tubes formed in matrigel by CMVECs were significantly more in AngII with Jagged1-siRNA group than in AngII without the siRNA group (Figure 6A-6C). Taken together, these results suggested that Jagged1 could contribute to the inhibitory effect of AngII on the angiogenetic responses of CMVECs through activating p53 (Figure 6D).

## Discussion

AngII is a major pathogenic factor during the development of cardiac remodeling and heart failure [10,20]. Inhibition of AngII and its type 1 receptor not only induce regression of cardiac hypertrophy but also improve cardiac dysfunctions [21,22]. Hypertrophy of cardiomyocytes and hyperplasia of fibroblasts have been known as two major important responses stimulated by AngII in the development of cardiac remodeling [22]. We here demonstrate the impairment of myocardial angiogenesis by AngII which may also contribute to the development of cardiac remodeling. It has been indicated that AngII-induced impairment of angiogenesis is secondary to the progress of cardiomyocyte hypertrophic response. However, we here provide the evidence for AngII to directly act on myocardial angiogenesis.

Sano M et al. observed that sustained pressure overload induced an accumulation of p53 in the heart that inhibited Hif-1α activity, thereby impairing cardiac angiogenesis and resulting in the transition from cardiac hypertrophy to heart failure [1,6]. The human p53 protein contains 393 amino acids and is comprised of several functional domains [1]. Under unstressed conditions, the p53 protein is generally present at a low concentration, presumably inactive as a transcription factor and diffusely distributed throughout the cell. In response to cellular stresses, p53 protein accumulates, frequently becomes extensively post-translationally modified and concentrates within the nucleus [23-26]. In the present study, we observed that AngII induced accumulation and phosphorylation of p53 in cytosol, and increases in phosphorylated p53 in nucleus both in cultured CMVECs and heart tissues. The phosphorylation of p53 in nucleus has been implicated in site-specific DNA binding [27] or binding to promoters of p53 target genes [2]. It has been reported to induce the downregulation of Hif-1α in nucleus [1]. Hif-1α is translocated to the nucleus where it induces expression of angiogenetic proteins, such as VEGF, against hypoxic injury [28,29]. In the present study, AngII exposure resulted in decreases of Hif-1α expression and VEGF protein production both in cutured CMVECs and in myocardium. In order to confirm the regulation of Hif-1α expression and VEGF production by p53 in AngII-induced impairment of angiogenesis, we used a specific p53 inhibitor PFT-α and observed that downregulation of nucleus Hif-1α expression and decrease of VEGF secretion from CMVECs induced by AngII were abrogated by the p53 inhibitor. These data collectively suggest that AngII induces cytosolic accumulation and phosphorylation of p53 and increases in phosphorylated p-53 in nucleus, leading to downregulation of nucleus Hif-1α, decrease of cellular VEGF production and impairment of myocardial angiogenesis.

Recent evidences reveal that angiogenetic factors regulate angiogenesis in concert with Notch signaling [30]. It has been reported that Notch ligands Jagged1 and Dll-4 have opposing effects on angiogenesis [7]. Jagged1 is a potent pro-angiogenetic regulator whereas Dll-4 inhibits angiogenesis of endothelial cells. We therefore examined the involvement of these two Notch ligands in the effect of AngII on angiogenesis. Our data revealed that AngII increased Jagged1 expression but it didn’t affect Dll-4 expression in cultured CMVECs, suggesting that Jagged1 rather than Dll-4 might participate in AngII-induced impairment of angiogenesis. To ask the role of the elevation of Jagged1 expression during the stimulation with AngII, we used siRNA method to knockdown Jagged1 in cultured CMVECs. Knocking-down of Jagged1 not only significantly inhibited the upregulation of p53 and the downregulation of Hif-1α but also improved the decrease of VEGF production and capillary-like tubes formation induced by AngII in cultured CMVECs, suggesting that Jagged1 contributes to the p53-regulated impairment of angiogenesis induced by AngII. To further examine the relationship between Jagged1 and p53, we observed the effect of p53 inhibitor on Jagged1 expression. Interestingly, inhibition of p53 by PFT-α greatly inhibited upregulation of Jagged1 stimulated by AngII both *in vitro* and *in vivo*, suggesting that p53 is required for AngII to stimulate Jagged1 expression. These results collectively indicate an regulation between p53 and Jagged1 in the setting of AngII-induced impairment of myocardial angiogenesis.

Benedito et al demonstrated that Jagged1 controls angiogenesis in the embryo and antagonizes the effects of Dll-4-mediated Notch signaling during sprouting angiogenesis in the neonatal retina by using endothelial-specific Jagged1 mutant or overexpressed mice [7]. It has also been indicated that angiogenetic sprouting in the tumor is tightly controlled by positive regulation of Jagged1 in edothelial and non-endothelial cells [31]. In the present study, Jagged1, as an anti-angiogenetic regulator, is involved in the inhibitory effect of AngII on myocardial angiogenesis, which is not consistent with the result of previous studies [7,31]. Although we do not know the reason for this discrepancy exactly, it might be explained by that Jagged1 exerts pro-angiogenetic or anti-angiogenetic effects dependently of cell types and stimulus.

Ligand, Deltalike or Jagged, binding to a Notch receptor results in its cleavage by a membrane-associated protease complex (γ-secretase) [32]. The released intracellular domain of the Notch receptor (ICN) is then translocated to the nucleus, and forms a complex with Rbpj and MAML transcription factor proteins, which directly activates downstream genes. In the mouse, multiple labs have shown that *Jag1-Notch-Rbpj* signaling is critically required during the second wave of lens fiber cell formation [19]. In the present study, Ang II upregulates Jagged1 expression and promotes the activation of RBPj in CMVECs which may contributes to Ang II-induced impairment of myocardial angiogenesis. Although p53 protein has been reported to regulate the activation of notch pathway at different levels in the expansion of mammary stem cells [33]. The regulation of p53 by Jagged1 in regulating cell function is unclear. Our data suggest that Ang II stimulates phosphorylation of p53 in nucleus of cultured CMVECs, and it binds promoters of p53 target genes, upregulates jagged1 expression, and activates notch signaling, in turn, the activation of notch signaling promotes the phosphorylation of p53, and then the activated p53 could bind to Rbpj to regulate the activation, finally resulting the downregulation of Hif-1α and VEGF, contributing to the impairment of Ang II on angiogenetic response. However, the exact explanation and the detailed mechanism of how p53 and Jagged1 interact in AngII-impaired angiogenetic functions of CMVECs should be explored.

In conclusion, AngII impairs cardiac angiogenesis by p53-dependent inhibition of HIF-1 which is regulated by Jagged1 in CMVECs. Thus, inhibition of p53 or Jagged1 in the heart may be a novel therapeutic strategy for improving the angiogenetic disorders induced by AngII. The strategy may be useful to preventing the transition from cardiac hypertrophy to heart failure.

## Supporting Information

Figure S1 **Analysis of capillary-tube formation of CMVECs.** Capillary-like tube formation was analyzed. CMVECs were seeded onto the matrigel in plates containing AngII (AngII) or PBS (Control). Eighteen hours later, Images of cultured CMVECs were taken at 50× magnification with a digital output camera attached to an inverted phase-contrast microscope (Leica, Germany), the formation of capillary-like tubes (white dots indicate) was observed and counted under an optical microscope. five random view-fields per well were counted to analyze the relative number of capillary-like tubes.
(TIF)

Figure S2 **Effects of p53 inhibitor on Hif-1 activity.** HUVECs were transfected with rRBPj-Luc construct together with β-gal expression plasmid for 48h, after 30min of PFT-α pretreatment, the cells were treated with Ang II for 24 hour and subjected for Luc assays. PFT-α: p53 inhibitor. Data are expressed as mean ±S.E.M. obtained from 3 independent experiments. ^**^ *p* < 0.01 *vs* DMSO or Saline; ^# #^ *p* < 0.01 *vs* AngII+DMSO.
(TIF)

Figure S3 **Effects of p53 inhibitor on AngII-induced-cardiac hypertrophy.** AngII (200 ng/kg/min) or saline was subcutaneously infused to mice for 2 weeks by Alzet micro-osmotic pumps. PFT-α (3.0 mg/kg) or DMSO was injected into mice intraperitoneally one day before AngII or saline infusion and then was injected in a similar dose once every 3 days during the infusion. Blood pressure. was measured by a noninvasive mice tail method. (**A**) Systolic (SBP). (**B**) diastolic BP (DBP). (**C**-**D**) Echocardiograghic analysis. LVPW.d, left ventricle posterial wall thickness at diastole phase; LVID.d, left ventricle internal dimension at diastole phase; (**E**) Heart weight to body weight ratio (HW/BW). (**F**) H-E staining of LV section. Representative photographs from LV section are shown (scale bar: 20 µm). (**G**) Quantification of cross section area (CSA) of cardiomyocytes. Data are expressed as mean ± S.E.M. obtained from 6 hearts. ^*^ *p* < 0.05 *vs* Saline; ^#^ *p* < 0.05 *vs* AngII+DMSO.
(TIF)

Figure S4 **Downregulation of Jagged1 by siRNA in HUVECs.** Three sequences of siRNA of Jagged1 (siRNA-Jagged1, 1 through 3) or scramble RNA were transfected to cultured HUVECs for 48 hours. Jagged1 expression was detected by Western blotting. β-Actin expression served as a loading control. Representative immunoblots are shown.
(TIF)

File S1 **Supplementary Methods.**
(DOC)
