# Supplementary figures and images for: Regulation of p53 by Jagged1 Contributes to Angiotensin II-Induced Impairment of Myocardial Angiogenesis
Source: PLoS One. 2013 Oct 3;8(10):e76529. doi: 10.1371/journal.pone.0076529 (PMC3789680; doi:10.1371/journal.pone.0076529)

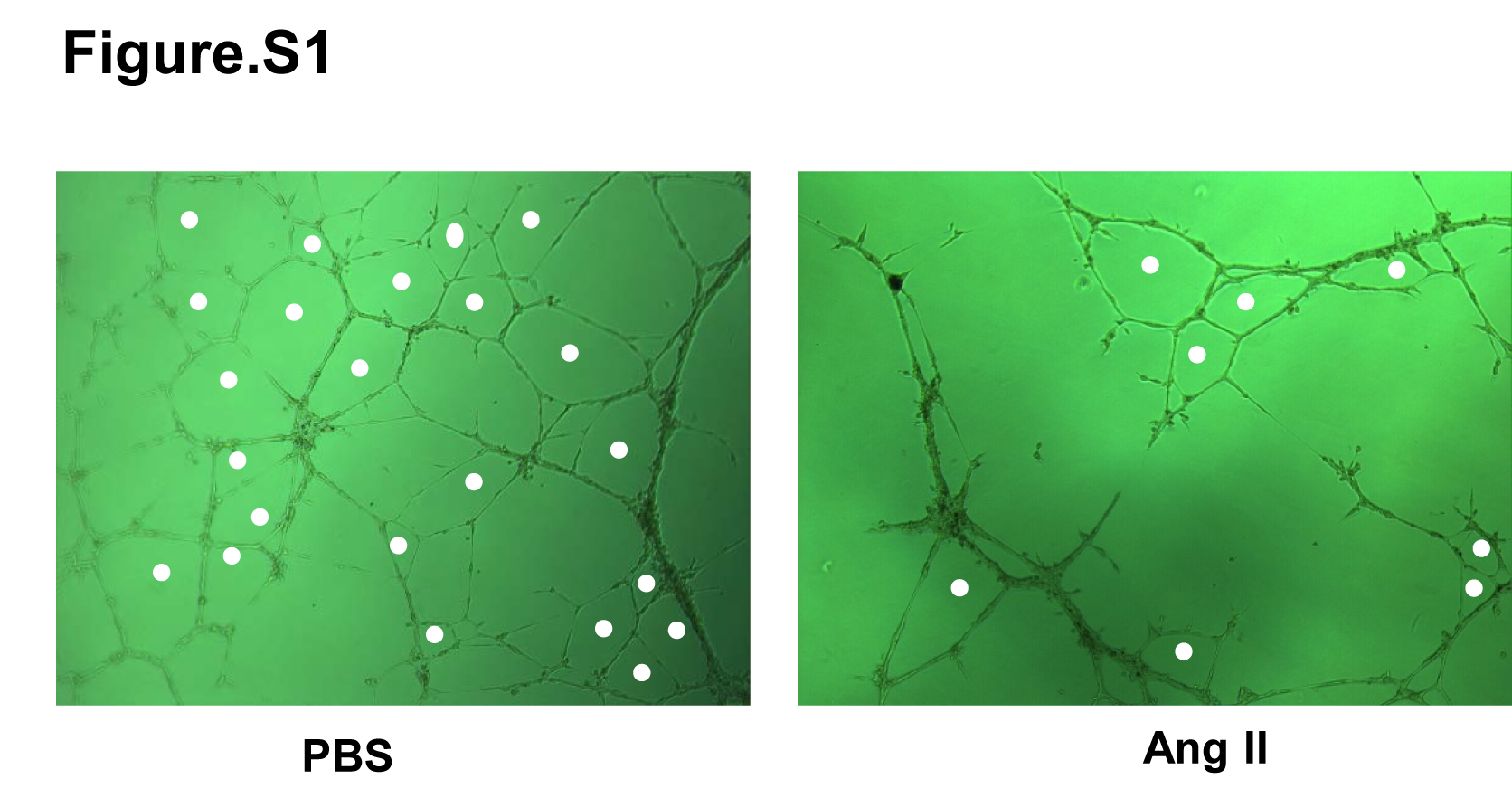

Supplement: Figure S1 — Analysis of capillary-tube formation of CMVECs. Capillary-like tube formation was analyzed. CMVECs were seeded onto the matrigel in plates containing AngII (AngII) or PBS (Control). Eighteen hours later, Images of cultured CMVECs were taken at 50× magnification with a digital output camera attached to an inverted phase-contrast microscope (Leica, Germany), the formation of capillary-like tubes (white dots indicate) was observed and counted under an optical microscope. five random view-fields per well were counted to analyze the relative number of capillary-like tubes. (TIF) [file pone.0076529.s001.tif]

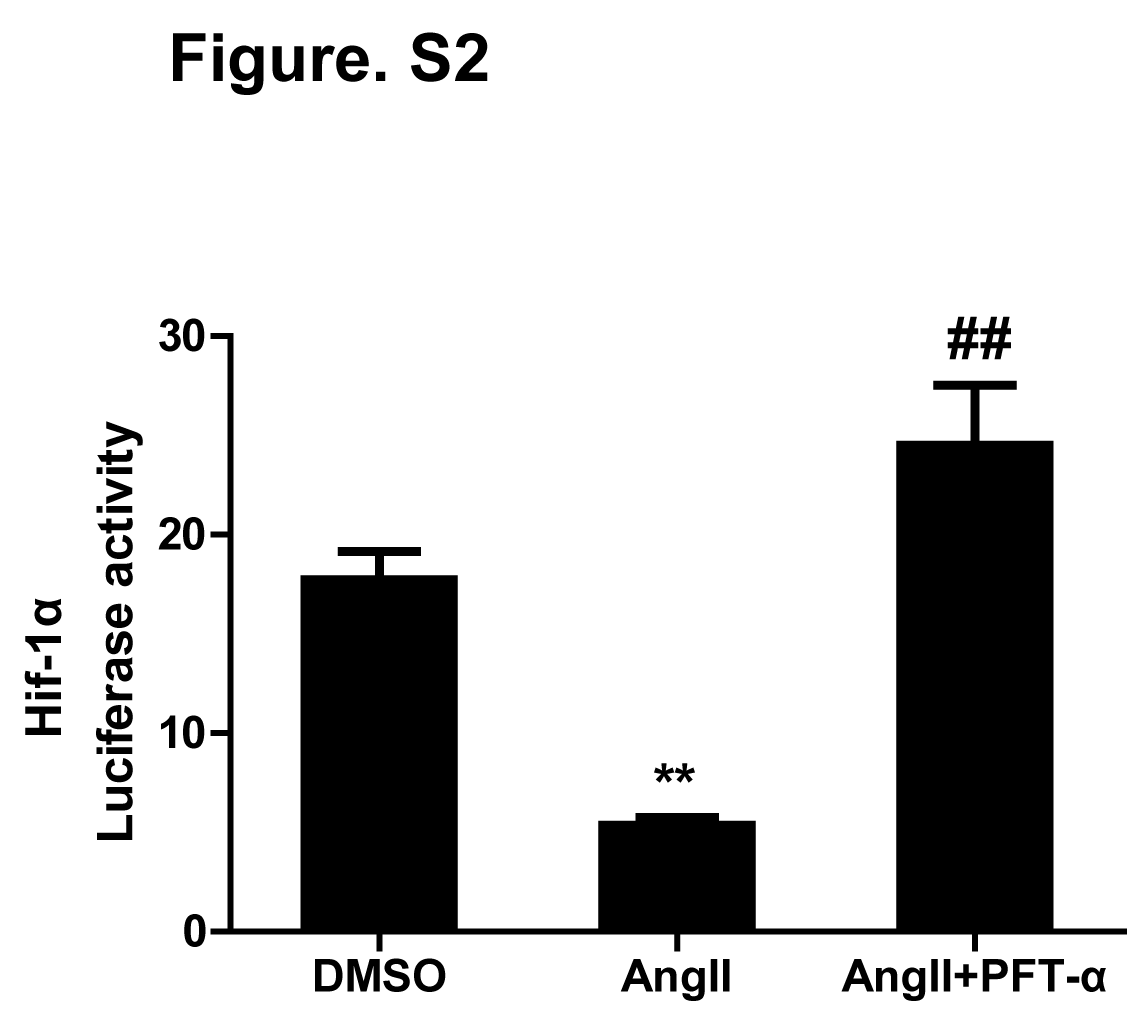

Supplement: Figure S2 — Effects of p53 inhibitor on Hif-1 activity. HUVECs were transfected with rRBPj-Luc construct together with β-gal expression plasmid for 48h, after 30min of PFT-α pretreatment, the cells were treated with Ang II for 24 hour and subjected for Luc assays. PFT-α: p53 inhibitor. Data are expressed as mean ±S.E.M. obtained from 3 independent experiments. ** p < 0.01 vs DMSO or Saline; # # p < 0.01 vs AngII+DMSO. (TIF) [file pone.0076529.s002.tif]

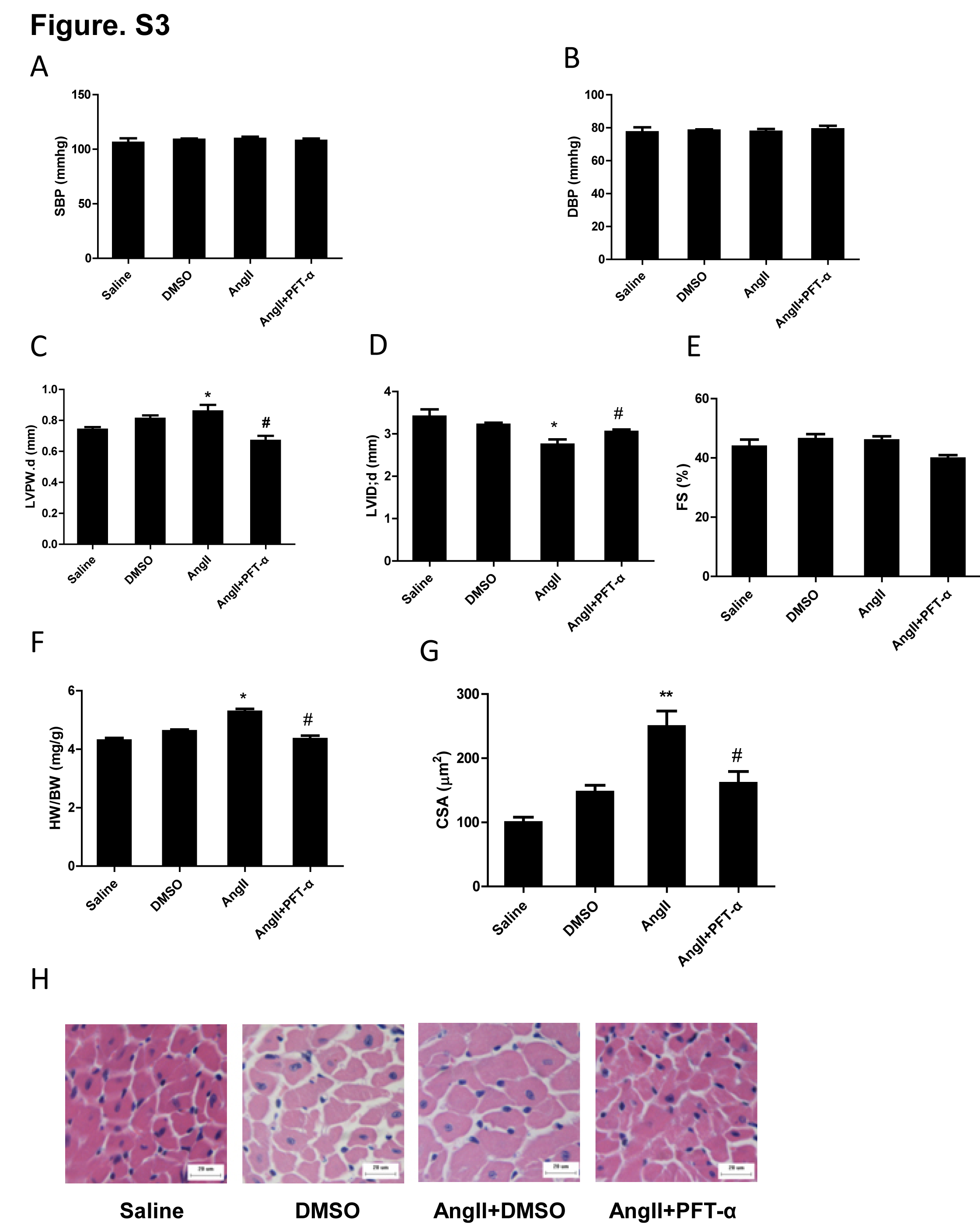

Supplement: Figure S3 — Effects of p53 inhibitor on AngII-induced-cardiac hypertrophy. AngII (200 ng/kg/min) or saline was subcutaneously infused to mice for 2 weeks by Alzet micro-osmotic pumps. PFT-α (3.0 mg/kg) or DMSO was injected into mice intraperitoneally one day before AngII or saline infusion and then was injected in a similar dose once every 3 days during the infusion. Blood pressure. was measured by a noninvasive mice tail method. (A) Systolic (SBP). (B) diastolic BP (DBP). (C-D) Echocardiograghic analysis. LVPW.d, left ventricle posterial wall thickness at diastole phase; LVID.d, left ventricle internal dimension at diastole phase; (E) Heart weight to body weight ratio (HW/BW). (F) H-E staining of LV section. Representative photographs from LV section are shown (scale bar: 20 µm). (G) Quantification of cross section area (CSA) of cardiomyocytes. Data are expressed as mean ± S.E.M. obtained from 6 hearts. * p < 0.05 vs Saline; # p < 0.05 vs AngII+DMSO. (TIF) [file pone.0076529.s003.tif]

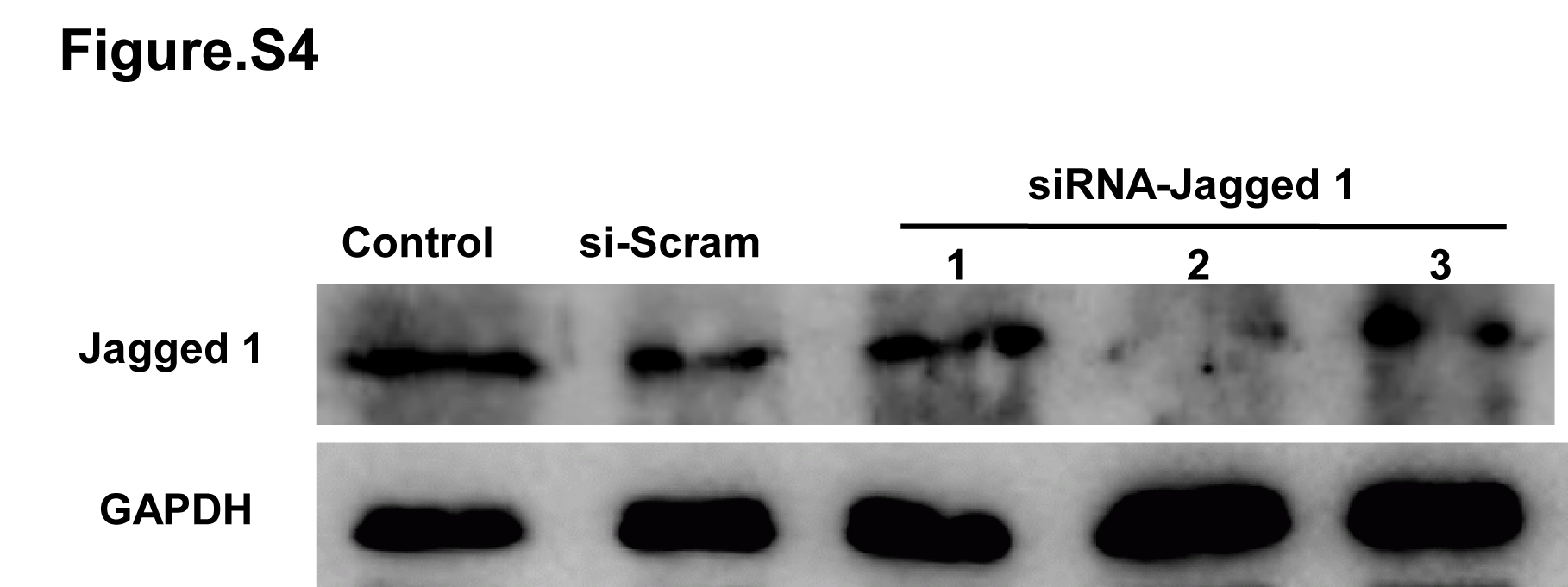

Supplement: Figure S4 — Downregulation of Jagged1 by siRNA in HUVECs. Three sequences of siRNA of Jagged1 (siRNA-Jagged1, 1 through 3) or scramble RNA were transfected to cultured HUVECs for 48 hours. Jagged1 expression was detected by Western blotting. β-Actin expression served as a loading control. Representative immunoblots are shown. (TIF) [file pone.0076529.s004.tif]
