# Supplementary material for: Regulation of p53 by Jagged1 Contributes to Angiotensin II-Induced Impairment of Myocardial Angiogenesis
Source: PLoS One. 2013 Oct 3;8(10):e76529. doi: 10.1371/journal.pone.0076529 (PMC3789680; doi:10.1371/journal.pone.0076529)
Supplement: File S1 — Supplementary Methods. (DOC) [file pone.0076529.s005.doc]

**Supplementary Materials**

**Supplementary Methods**

**Echocardiography and blood pressure measurement**

Transthoratic echocardiography was performed using 30MHz high frequency scanhead (VisualSonics Vevo770, VisualSonics Inc. Toronto, Canada). All measurements were averaged for five consecutive cardiac cycles and were carried out by three experienced technicians who were unaware of the identities of the respective experimental groups. Noninvasive blood pressure (BP) (Kent Scientific) of the mice tail was measured before the performance of transthoratic echocardiography.

**Histological analysis**

Excised hearts were perfused with PBS and fixed in 10% formalin for histological analysis. Paraffin embedded hearts were sectioned at 4 μm thickness and stained with hematoxylin and eosin (H-E). Photographs of LV for each sample were taken and measured by a video camera (Leica Qwin 3) attached to microscope. CSA of cardiomyocytes was measured in 20 different randomly chosen points from each cross section of LV wall. Five sections were analyzed for each heart.
